# Impact of Antiarrhythmic Drugs on the Outcome of Short QT Syndrome

**DOI:** 10.3389/fphar.2019.00771

**Published:** 2019-08-02

**Authors:** Ibrahim El-Battrawy, Johanna Besler, Xin Li, Huan Lan, Zhihan Zhao, Volker Liebe, Rainer Schimpf, Siegfried Lang, Christian Wolpert, Xiaobo Zhou, Ibrahim Akin, Martin Borggrefe

**Affiliations:** ^1^First Department of Medicine, Faculty of Medicine, University Medical Centre Mannheim (UMM), University of Heidelberg, Mannheim, Germany; ^2^DZHK (German Center for Cardiovascular Research), Partner Site, Heidelberg-Mannheim, Mannheim, Germany

**Keywords:** short QT syndrome, sudden cardiac arrest, channelopathy, congenital disease, arrhythmia

## Abstract

Short QT syndrome (SQTS) is associated with sudden cardiac arrest. There are limited data on the impact of antiarrhythmic drugs on the outcome of SQTS.

**Materials and Methods:** We studied data that describe the clinical outcome of 62 SQTS patients treated with antiarrhythmic drugs, who were recruited from a pool of patients diagnosed in our institution and also from known databases after a systematic search of the published literature.

**Results:** Sixty-two SQTS patients treated with antiarrhythmic drugs were followed up over a median timeframe of 5.6 years (interquartile range 1.6–7.7 years). Six patients, in particular, received multiple drugs as a combination. Of the 55 patients treated with hydroquinidine (HQ), long-term prophylaxis was documented in 41 patients. Fourteen patients stopped treatment due to the following reasons: gastrointestinal intolerance (n = 4), poor compliance (n = 8), and no QTc prolongation (n = 2). Of the 41 patients treated with HQ, the QTc interval increased from 313.5 ± 17.2 to 380.1 ± 21.2 ms. Thirteen of the 41 patients suffered from at least one or more ventricular tachyarrhythmias (VAs) before HQ initiation. VAs are reduced in incidence after HQ treatment (13/41: 31% versus 3/41: 7.3%, p < 0.001).

**Conclusion:** HQ increases the corrected QT interval and prevents VAs in the majority of the patients in this cohort. HQ is safe for use in SQTS patients, particularly due to its low rate of side effects. Other antiarrhythmic drugs might be useful, but the data justifying their use are sparse.

## Introduction

Short QT syndrome (SQTS) is a rare channelopathy, and patients affected with this disorder are predisposed to a higher risk of developing ventricular tachyarrhythmias (VAs; Giustetto et al., 2011; Mazzanti et al., 2014). Although significant progress has been made in the last decade to better understand this channelopathy, there are still significant challenges that physicians face in its management. Since its first description in 2000 (Gussak et al., 2000), causative mutations have been identified in eight different genes: SQTS1 to SQTS3 are associated with gain-of-function mutations in potassium channels, whereas SQTS4 to SQTS6 are caused by loss-of-function of calcium channels, demonstrating some overlap with Brugada syndrome. A loss-of-function mutation in SCN5A was detected in a patient with SQTS and Brugada syndrome (Hong et al., 2012). Although there are no conclusive data regarding the association of this variant with SQTS, it was described as SQTS7. Recently, a mutation in the cardiac Cl/HCO_3_ exchanger AE3 was found to also cause SQTS and described as SQTS8 (Thorsen et al., 2017). Recently, clinical guidelines have recommended hydroquinidine (HQ) to treat SQTS patients (Priori et al., 2015b). Although different antiarrhythmic drugs have been tested in a small series of SQTS patients (Schimpf et al., 2007;Zhao et al., 2019), there are few data describing the long-term outcome of treated patients.

A recently published report by our research group showed a significant reduction of arrhythmia-like events in human cardiomyocytes from induced pluripotent stem cells established from patients with SQTS1 in the presence of N588K mutation after incubation of cells with 10 µM quinidine (El-Battrawy et al., 2018).

The aim of the present study was to consolidate our experience treating SQTS patients with reports from other groups using a systematic method of review.

## Methods

We defined SQTS according to the Gollob criteria published in 2011 and the ESC criteria published in 2015 (Gollob et al., 2011; Priori et al., 2015b). Syncope was described as the transient loss of consciousness in the absence of other causes. An arrhythmic event was defined as a documented ventricular fibrillation (VF)/ventricular tachycardia (VT).

Of the 10 families admitted to our hospital with suspected SQTS, 7 families fulfilled the prescribed SQTS criteria. The family screening cascade further permitted the diagnosis of SQTS in 9 affected relatives. The QT interval was measured using the tangent method in the precordial lead presenting the highest T-wave amplitude in V2 or V3. The diagnosis of SQTS was independently reviewed by two experienced blinded cardiologists. DNA sequencing using next-generation sequencing of affected genes (*KCNH2*, *KCNQ1*, *KCNJ2*, *CACNA1C*, *CACNB2*, and *CACNA2D1*) for SQTS1 to SQTS6 was performed for the purpose of genetic screening and then analyzed for results.

The study was approved by the local ethics committee of the University Hospital Mannheim, University of Heidelberg, Germany. A written informed consent was not required by the local ethics committee due to the observational and retrospective characteristics of the study.

### Systematic Literature Review

**Figure 1:**
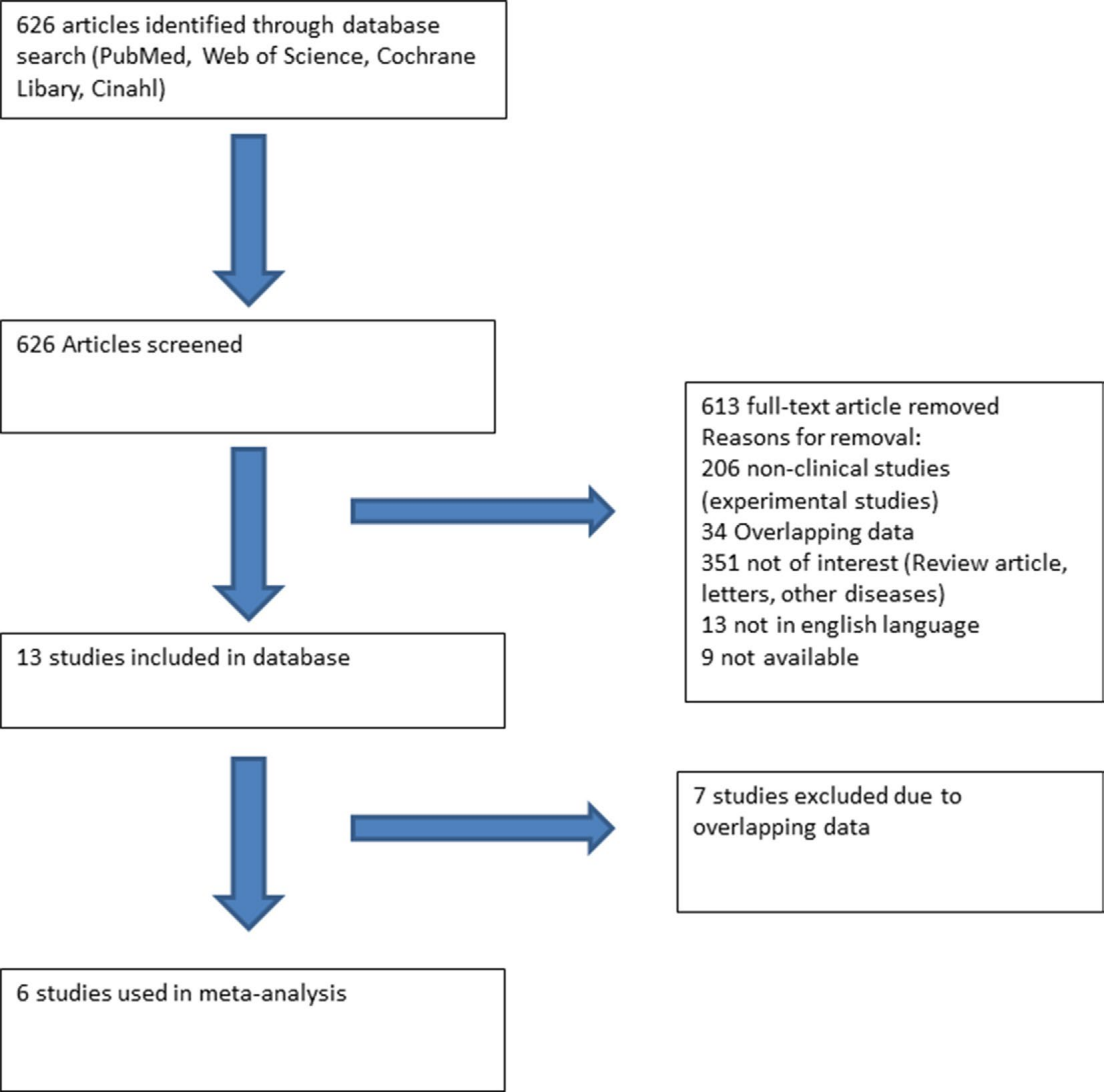
Flow chart presenting the systematic literature review using PubMed, Web of Science, Cochrane Library, and Cinahl. Six studies reporting the use and outcome of drugs in SQTS patients were included.

A literature search (PubMed, Web of Science, Cochrane Library, and Cinahl) with limits defining the publication dates up to 2018, use of the English language, and studies with human subjects was performed. Clinical studies earmarked included SQTS patients with well-described therapeutic lines of management (**Figure 1**). All these studies were based on data recorded from electrocardiograms (ECGs) and principally excluded patients with structural heart disease. Additionally, case reports and studies not reporting the outcome of antiarrhythmic drug use were excluded.

### Statistics

Data are presented as mean ± SD for continuous variables with a normal distribution, median [interquartile range (IQR)] for continuous variables with a nonnormal distribution, and frequency (%) for categorical variables. Kolmogorov-Smirnov test was used to assess normal distribution. Student’s t-test and Mann-Whitney U-test were used to compare continuous variables with normal and nonnormal distributions, respectively. Chi-square test or Fisher’s exact test was used to compare categorical variables.

## Results

### Demographics of Seven SQTS Families

The baseline characteristics of SQTS patients are illustrated in **Table 1**. Seven of the 10 SQTS families (n = 17 patients) fulfilled the criteria for SQTS and were retrospectively followed. Majority of the patients presented symptoms, including syncope (n = 5), palpitation (n = 8), and atrial fibrillation and atrial flutter (n = 9). Aborted sudden cardiac arrest (SCA) was documented in 2 (12%) patients with a confirmed short QT interval at admission and in 1 patient after receiving implantable cardioverter defibrillator (ICD) implantation. Eight (47%) patients were discharged with antiarrhythmic drugs, including HQ (n = 6, 35%) and bisoprolol (n = 2, 12%). Five (29%) patients underwent an ICD implantation, 3 of them were based on a high risk of SCA due to the abbreviated QTc interval of <300 ms and the presence of recurrent familial SCA.

**Table 1:** Baseline characteristics of all SQTS cohorts (treated with or without drug).

Study	(El-Battrawy et al., 2018)	(Bun et al., 2012)	(Mazzanti et al., 2017)	(Guistetto et al., 2011)	(Villafane et al., 2013)	(Suzuki et al., 2014)	(Guistetto et al., 2015)
**Variables**	**n = 17**	**n = 1**	**n = 17**	**n = 53**	**n = 25**	**n = 1**	**n = 3**
Age, median	34	28	29	26	15	10	21
Male, n (%)	8 (48%)	1 (100%)	14 (82%)	40 (75%)	21 (84%)	1 (100%)	1 (33%)
Female, n (%)	9 (52%)	0 (0%)	3 (18%)	13 (25%)	4 (16%)	0 (0%)	2 (66%)
**Symptoms, n (%)**							
Syncope	5 (29%)	0 (0%)	0 (0%)	8 (15%)	4 (16%)	0 (0%)	0 (0%)
Palpitation	8 (47%)	0 (0%)	0 (0%)	13 (24%)	4 (16%)	0 (0%)	0 (0%)
SCA	2 (12%)	1 (100%)	6 (35%)	18 (34%)	6 (24%)	0 (0%)	1 (33%)
Atrial flutter	2 (11%)	0 (0%)	0 (0%)	0(0%)	0 (0%)	0 (0%)	0 (0%)
Atrial fibrillation	7 (41%)	0 (0%)	0(0%)	0 (0%)	4 (16%)	0 (0%)	0 (0%)
nsVT	0 (0%)	0 (0%)	0 (0%)	0 (0%)	0 (0%)	0 (0%)	2 (66%)
Ventricular ectopy	0 (0%)	0 (%)	0 (= %)	6 (11%)	0(%)	0(0%)	1(33%)
Asymptomatic	0 (0%)	0 (0%)	11 (64%)	0 (0%)	0 (0%)	1 (100%)	0 (0%)
**Medical treatment, n (%)**							
Yes	8 (47%)	1 (100%)	17 (100%)	22 (41%)	10 (40%)	1 (100%)	3 (100%)
**ICD implantation, n (%)**							
yes	5 (29%)	1 (100%)	9 (53%)	24(45%)	11 (44%)	0 (0%)	1 (33%)
**Genetic screening, n (%)**							
CaCNB2B	6 (35%)	0 (0%)	0 (0%)	2 families	0 (0%)	0 (0%)	0 (0%)
CaCNA1C	3 (17%)	0 (0%)	0 (0%)	0 (0%)	0 (0%)	0 (0%)	0 (0%)
KCNH2	4 (23%)	0 (0%)	0 (0%)	11(20%)	2 (8%)	1 (100%)	3 (100%)
KCNQ1	0 (0%)	0 (0%)	1 (6%)	0 (0%)	1 (4%)	0 (0%)	0 (0%)
KCNJ2	0 (0%)	0 (0%)	1 (6%)	0 (0%)	2 (8%)	0 (0%)	0 (0%)

### Systematic Literature Review

#### Eligible Studies

We conducted a systematic literature review to identify SQTS patients and their outcomes after antiarrhythmic drug use. A total of six studies/cases were deemed eligible for follow-up. The patient pool from our center included 8 patients, which were consequently added to this analysis, thus leading to a total of 62 SQTS patients available for this study (Giustetto et al., 2011; Bun et al., 2012; Villafane et al., 2013; Suzuki et al., 2014; Giustetto et al., 2015; Mazzanti et al., 2017;El-Battrawy et al., 2018). The baseline characteristics of the included studies are illustrated in **Table 1**.

### Tested Drugs and Influence of the QTc

Different drugs were tested: HQ (n = 55), bisoprolol (n = 2), flecainide (n = 1), sotalol (n = 5), sotalol plus propafenone (n = 1), propafenone plus digoxin (n = 1), dofetilide plus digoxin (n = 1), disopyramide (n = 3), amiodarone (n = 1), and amiodarone plus metoprolol (n = 1). HQ prolonged the QTc interval in 52 of 55 (94%) patients (**Table 3**). Other treatment regimens, including sotalol, sotalol plus propafenone, flecainide, amiodarone, and bisoprolol failed to increase the QTc interval in treated patients. However, digoxin plus dofetilide, amiodarone plus metoprolol, and disopyramide with the higher dosage (400 mg/day) presented a QTc prolongation in ECG (**Table 2** and **Table S1**).

**Table 2:** Drug treatment strategy in SQTS patients (of **Table 1**) and side effects.

Author	(El-Battrawy et al., 2018)	(Bun et al., 2012)	(Mazzanti et al., 2017)	(Guistetto et al., 2011)	(Villafane et al., 2013)	(Suzuki et al., 2014)	(Guistetto et al., 2015)
Patients on antiarrhythmic drugs on discharge, n (%)	8	1	17	22	10	1	3
Age, median (years)	34	28	29	26	15	10	21
HQ, n	6	1	17	22	5	1	3
Flecainide, n	0	0	0	0	1	0	0
Sotalol, n	0	0	0	3	0	0	2
Sotalol plus propafenone, n	0	0	0	0	1	0	0
Propafenone plus digoxin, n	0	0	0	0	1	0	0
Dofetilide plus digoxin, n	0	0	0	0	1	0	0
Disopyramide, n	0	0	0	3	0	0	0
Bisoprolol, n	2	0	0	0	0	0	0
Amiodarone, n	0	0	0	1	0	0	0
Amiodarone plus metoprolol	0	0	0	1	0	0	0
Dosage HQ (mg) daily, mean	916.66	600	584	870	—	20 mg/kg	666.66
Side effects of HQ and stopped treatment	n = 2 poor compliance	none	n = 2 gastrointestinal intolerance	n = 6 poor compliance, n = 2 no effect on QTc, n = 2 gastrointestinal intolerance	—	none	
QTc before HQ (ms)	318 ± 29 (n = 6)	320	331 ± 3	307 ± 20 |(n = 18)	304 ± 42 (n = 25)	283	332 ± 23
QTc after HQ (ms)	404 ± 46 (n = 6)	383	391 ± 9	384 ± 39 (n = 18)	—	341	378 ± 33
EPU before HQ	n = 3 inducible arrhythmias	NA	NA	n = 8 EPU done before and after drug	—		n = 2 short atrial and ventricular refractory periods
EPU after HQ	n = 3 no inducible arrhythmias anymore	NA	NA	n = 8 none inducible VF	—		NA
Follow-up (months) days	4860	180	2130	1920	2130	—	708

—, not documented; NA, not available.

### Impact of HQ on the Outcome and Side Effects

**Figure 2:**
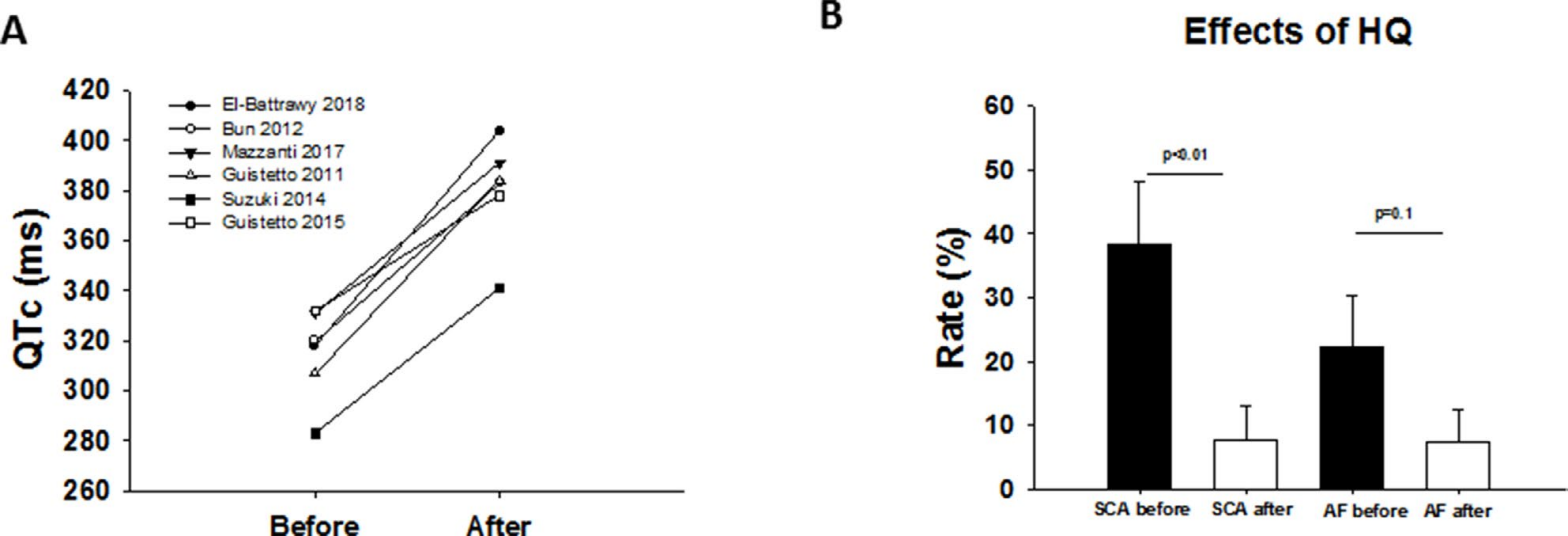
**(A)** Overview showing the QTc interval before and after drug treatment (HQ) in different studies. **(B)** Effect of HQ on VAs and atrial fibrillation rate.

Of the 55 SQTS patients treated by HQ, only 41 patients reported long-term use after a median follow-up time of 5.6 years (IQR 0.5–13.5 years). The QTc interval was increased in all reported studies, except in two cases (**Figure 2A**). Thirteen patients suffered from at least one or more VA event before HQ initiation. VAs have been dropped after HQ treatment (31% versus 7.3%, p < 0.001; **Figure 2B**). There was no significant change in the rates of atrial fibrillation (**Figure 2B**). Fourteen patients stopped treatment due to the following reasons: gastrointestinal intolerance (n = 4), poor compliance (n = 8), and no QTc prolongation (n = 2).

### Electrophysiological Studies Before and After HQ Treatment

**Figure 3:**
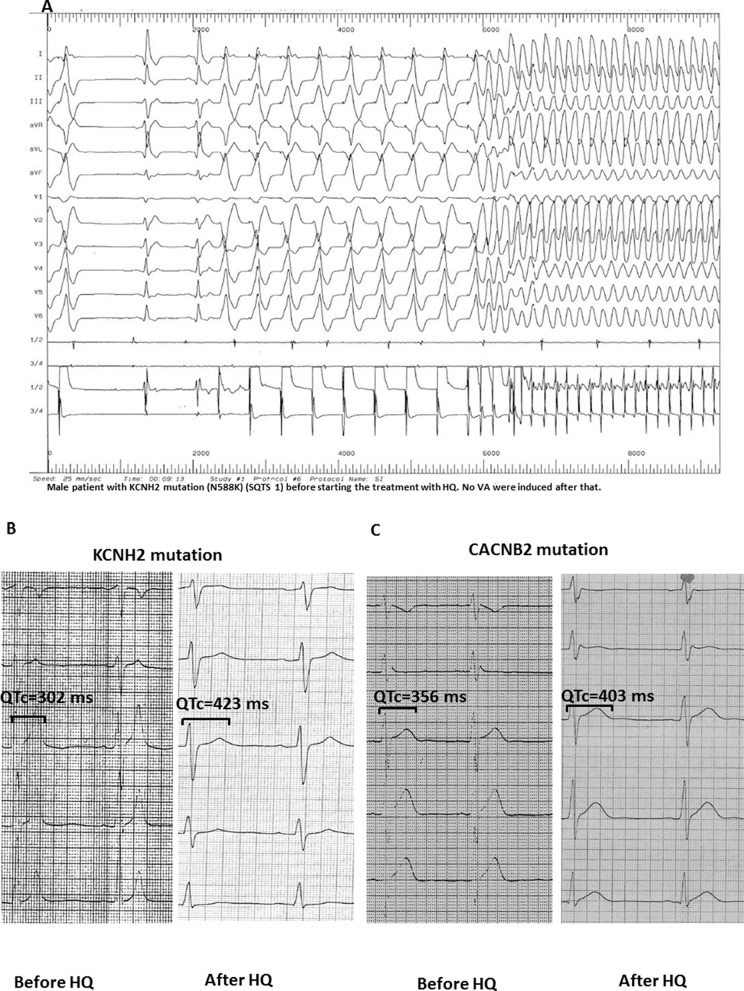
**(A)** Induction of ventricular flutter (CL 150 ms) in a patient with KCNH2 mutation (N588K; SQTS1) before starting HQ treatment. **(B** and **C)** After the application of HQ, the QTc interval (corrected by Bazett formula) was significantly increased in SQTS1 and SQTS5 patients without induction of arrhythmias in the electrophysiology study.

In two clinical studies, an electrophysiological study was conducted before and after HQ treatment (n = 11; **Figure 3A**). In these cases, an VF/VT was found to be inducible before treatment. However, these could not be reproduced once treatment was initiated.

#### Detailed Analysis of the Baseline Characteristics of HQ Treatment

**Figure 4:**
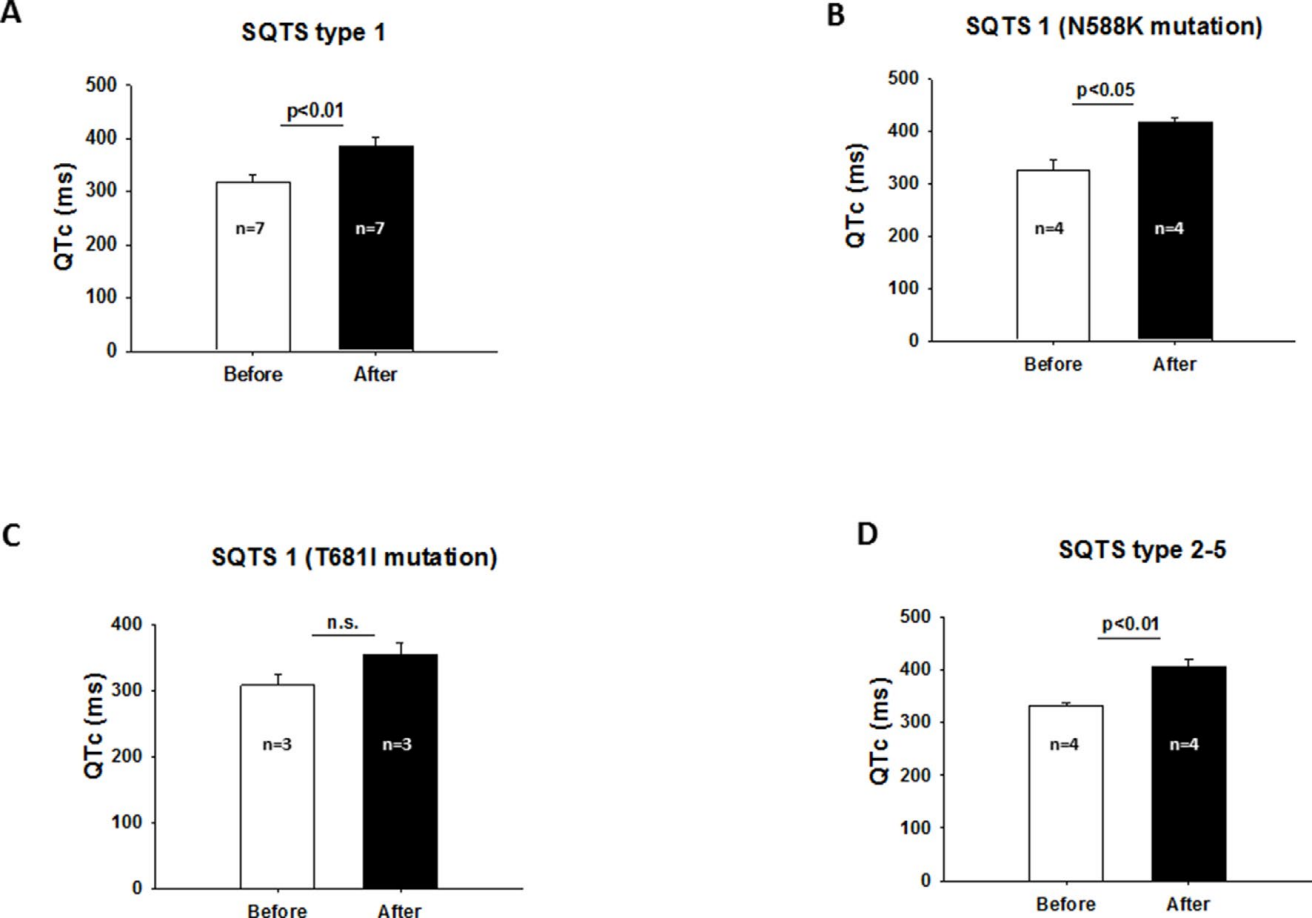
** (A)** Increased QTc interval after HQ treatment in the presence of SQTS1. **(B)** QTc interval was significantly prolonged in the presence of N588K mutation of KCNH2 after HQ treatment. **(C)** QTc interval was not significantly prolonged after HQ use in the presence of KCNH2 mutation in T618I. **(D)** Effect of HQ on QTc interval in the presence of SQTS2 to SQTS5.

**Table 3** includes all studies with a detailed description of baseline characteristics such as gender, QTc, gene mutation, and symptoms including atrial fibrillation and VAs of patients treated with HQ. Two studies (Giustetto et al., 2011; Villafane et al., 2013) were excluded due to the absence of details of every patient. Using cases with available information about the SQTS-related gene, the outcome data, a detailed analysis of the mutation-specific effects of HQ on QTc and arrhythmias was performed. In the mutation group, seven patients had a mutation in KCNH2 [either N588K (n = 4) or T618I (n = 3)] and four other patients had mutations in CACNA1C [G490R (n = 1)], CACNB2B [C1422T (n = 1)], KCNQ1 [R259H (n = 1)], and KCNJ2 [D172N (n = 1)]. In all patients, except two patients with KCNH2 mutation in T618I, life-threatening arrhythmias were suppressed. Although the QTc interval of the whole population was significantly increased, a detailed grouping of patients regarding gene mutations showed some differences. For example, although the QTc was prolonged in the presence of KCNH2 mutations (**Figure 4A**), this prolongation was only significant in the presence of N588K mutation (**Figure 4B**) but not in the presence of T618I mutation (**Figure 4C**). In patients with SQTS2 to SQTS5 (mutations in CACNA1C, CACNB2B, KCNQ1, and KCNJ2), QTc was also significantly prolonged by HQ (**Figure 4D**). Taking all these data together, it seems like that patients with T618I mutation of KCNH2 does not respond to HQ treatment.

**Table 3:** Details of different SQTS types treated by HQ.

Case	Author	Sex	Gene	Mutation	Current	Symptoms	Arrhythmias	Type of SQTS	Drugs	Side effects	Outcome	QTc before drug (ms)	QTc on drugs (ms)	Follow-up days
1	(El-Battrawy et al., 2018)	F	KCNH2	N588K	I_Kr_	Palpitation	Atrial fibrillation	SQTS1	HQ	None	No VA and no inducible arrhythmias in EP study anymore	351	435	5564
2		F	KCNH2	N588K	I_Kr_	None	None	SQTS1	HQ	None	No VA and no inducible arrhythmias in EP study	268	400	5427
3		M	KCNH2	N588K	I_Kr_	Syncope	Atrial fibrillation	SQTS1	HQ	None	No shocks while on HQ more	329	—	4572
4		M	CaCNA1C	G490R	I_Ca-L_	Palpitation	Atrial fibrillation	SQTS4	HQ	None	HQ treatment stopped (no compliance)	347	446 (HQ)	4720
5		M	CaCNB2B	C1422T	I_Ca-L_	Syncope SCD	Atrial fibrillation Atrial flutter	SQTS5	HQ	None	Two inappropriate shocks while with HQ	329	373	5756
6		M	—	—	—	Palpitation	Atrial fibrillation Atrial flutter	SQTS	HQ+Ve	None	HQ treatment stopped (no compliance)	326	449 (HQ+Ve)	5195
7	(Bun et al., 2012)	M	—	—	—	SCD	VF	SQTS	HQ	None	No recurrence of VA	320	—	180
8	(Mazzanti et al., 2017)	M	—	—	—	Syncope	None	SQTS	HQ	None	No LTA after initiating HQ	312	398	2310
9		M	—	—	—	None	None	SQTS	HQ	None	No VA	314	350	
10		F	KCNQ1	R259H	I_Ks_	SCD	None	SQTS2	HQ	None	No VA	316	405	
11		M	—	—	—	None	None	SQTS	HQ	None	No VA	321	412	
12		M	—	—	—	SCD	None	SQTS	HQ	None	No VA	324	418	
13		F	—	—	—	None	None	SQTS	HQ	None	No VA	326	356	
14		M	KCNJ2	D172N	I_k1_	None	None	SQTS3	HQ	None	No VA	332	396	
15		M	—	—	—	Syncope	None	SQTS	HQ	None	No VA	337	388	
16		M	—	—	—	None	None	SQTS	HQ	None	No VA	338	390	
17		M	—	—	—	SCD	None	SQTS	HQ	None	No VA	338	398	
18		M	—	—	—	None	None	SQTSn	HQ	None	No VA	339	358	
19		M	—	—	—	None	None	SQTS	HQ	None	No VA	340	382	
20		M	—	—	—	SCD	None	SQTS	HQ	None	No VA	344	413	
21		M	—	—	—	SCD	None	SQTS	HQ	None	No VA	348	390	
22		M	—	—	—	SCD	None	SQTS	HQ	None	No VA	351	410	
23	(Suzuki et al., 2014)	M	KCNH2	N588K	I_Kr_	None	None	SQTS1	HQ	None	—	283	341	—
24	(Guistetto et al., 2015)	M	KCNH2	T618I	I_Kr_	None	Ventricular ectopy	SQTS1	HQ	None	Ventricular ectopy has been suppressed	300	333	1770
25		F	KCNH2	T618I	I_Kr_	None	None	SQTS1	HQ	None	Loops recorder detected one slow nsVT	340	389	1770
26		F	KCNH2	T618I	I_Kr_	SCD	nsVT	SQTS1	HQ	None	Still runs of nsVT but slower on HQ	355	411	1770

EP, electrophysiology; —, not documented; SCD, sudden cardiac death; Bis VE, verapamil; ME, metoprolol; Bi, bisoprolol.

#### Detailed Analysis of the Baseline Characteristics of SQTS Treatment With Other Drugs

All studies with a detailed description of the baseline characteristics of SQTS treatment with other drugs are summarized in **Table S1**. Digoxin plus dofetilide and amiodarone plus metoprolol showed efficacy for preventing arrhythmias in SQTS patients.

## Discussion

We have described the short- and long-term impact of antiarrhythmic drugs in SQTS patients from our hospital and those from a systematic literature review and can ascertain the following: i) HQ is safe for use among SQTS patients; ii) HQ significantly reduces VAs and consequently ICD shocks; iii) the benefit of HQ might be higher in SQTS patients of unknown origin; and iv) sotalol, sotalol plus propafenone, flecainide, amiodarone, disopyramide, and bisoprolol failed to show beneficial effects on SQTS.

ICD implantation has been recommended for SQTS patients (Priori et al., 2015a). However, ICD implantation is associated with adverse events and survivors of VA events are at high risk of recurrence several years later with risk of repeated ICD shocks. Therefore, a medical approach needs to be considered for SQTS patients.

In our center, the medical line of management was pursued among eight affected patients. HQ was initiated in six patients, with poor compliance in two of these patients. Two patients, who were started on beta-blockers, did not show changes of the QTc interval.

A systematic review of the literature showed that HQ use significantly reduced the recurrence of VAs. Additionally, in electrophysiological studies, VT/VF could not be induced after HQ use (11 of 11 patients). About 7% of the cases in the patient cohort initiated on HQ reported side effects such as gastrointestinal intolerance, leading to the termination of treatment. Importantly, HQ did not induce arrhythmias. Sotalol was used in five SQTS patients with no effect on the QTc interval.

HQ is a multichannel blocker. Due to the block of the I_Kr_, it is able to prolong the action potential duration and consequently the QTc interval. However, sotalol and amiodarone, which are also I_Kr_ blockers and prototypical QT-prolonging drugs, were ineffective in patients with SQTS1. Of note, whereas amiodarone is a multichannel blocker, sotalol is a beta-blocker. It has been suggested that drug effects in SQTS might be based on the type of mutation. Some studies have demonstrated that the site mutation of N588K in HERG channel attenuates the inactivation of the channel and thus increases the whole-cell channel current at physiological voltages by shifting rightward the voltage dependence of inactivation (Cordeiro et al., 2005; Mcpate et al., 2005). Strikingly, this inactivation defect reduces strongly the sensitivity of the channel to some I_Kr_ blockers such as sotalol and E-4031, which have the highest effect in the inactivated state of HERG and are therefore less effective in inhibiting the channel currents (Vitali Serdoz et al., 2019). HQ has a similar effect in the open and inactivated states of HERG channels and is therefore effective in inhibiting I_Kr_ and treating SQTS1 patients.

Another study demonstrated that double mutations in HERG channel further enhance the impairment of the channel gating and channel response to drugs (Mcpate et al., 2008). In the presence of N588K and S631A mutations, the channel inactivation and the effect of HQ were more strongly attenuated compared to N588K or S631A mutation alone. Taken together, mutations in an ion channel can influence both channel gating and drug effects. In our present systematic review, two of three patients carrying a KCNH2 mutation in T618I recurrently suffered from VAs despite HQ treatment and the QTc interval was not significantly increased. Therefore, it might be speculated that T618I mutation impacts the sensitivity of the channel to HQ and therefore its antiarrhythmic effect in SQTS patients, although the effects of HQ on I_Kr_ mediated by HERG channels with T618I needs to be examined.

This study is consistent with recently published data by our research group studying human cardiomyocytes from induced pluripotent stem cells derived from SQTS donors (El-Battrawy et al., 2018). Our *in vitro* study showed that quinidine led to a prolongation of the action potential duration, consistent with a prolongation of the QTc interval, leading to a significant reduction in arrhythmia-like events among SQTS patients, whereas other antiarrhythmic drugs such as sotalol and metoprolol did not show any effect.

As the prevalence of SQTS is low (Bjerregaard, 2018), there are little data documenting the experience of drug treatments in this scenario. The documented adverse events in 7% of patients treated with HQ may provide an impetus to test other drugs. Our systematic literature analysis showed serial cases of use of treatment regimens such as digoxin plus dofetilide, amiodarone plus metoprolol, and disopyramide, which caused QTc prolongation (Gaita et al., 2004; Wolpert et al., 2005; Mizobuchi et al., 2008; Giustetto et al., 2011). However, these data need to be confirmed in the laboratory followed by testing in a clinical scenario involving a higher number of patients.

## Conclusion

HQ, an off-label drug, increases the QTc interval and reduces VA events in SQTS patients. Due to the adverse effects and ineffectiveness of HQ in some patients, searching for other antiarrhythmic drugs is required.

## Study Limitations

First, indications for HQ use have not been homogeneous throughout the study. HQ use was dependent on the experience of the clinical team and no control group has been identified. Second, these studies were not randomized, and no comparative analyses were drawn to other drugs. Third, it is not excluded that SQTS-related genes were not screened; therefore, the SQTS-related gene is underestimated. Finally, although this study represents the largest data accumulation among the recently published reports, it may still not be sufficient to fully understand the true outcome of SQTS patients initiated on HQ treatment. A further significant limitation is that QT interval measurement using the tangent method in the precordial lead presenting the highest T-wave amplitude in V2 or V3 might be biased by interobserver variability and may not be consistent with those data from the our institution.

## Author Contributions

IE-B, XL, ZZ, JB, MB, and IA contributed to the conception and design of the work. RS, SL, and CW contributed to the acquisition, analysis, and interpretation of data for the work. IE-L, MB and IA drafted the manuscript. XZ, VL, RS, and CW critically revised the manuscript. All gave final approval and agree to be accountable for all aspects of work ensuring integrity and accuracy.

## Conflict of Interest Statement

The authors declare that the research was conducted in the absence of any commercial or financial relationships that could be construed as a potential conflict of interest.
