# Supplementary material for: Impact of Antiarrhythmic Drugs on the Outcome of Short QT Syndrome
Source: Front Pharmacol. 2019 Aug 2;10:771. doi: 10.3389/fphar.2019.00771 (PMC6688193; doi:10.3389/fphar.2019.00771)
Supplement: Supplementary file 1 [file Table_1.docx]

| **Case** | **Author** | **Sex** | **Gene** | **Mutation** | **Current** | **Symptoms** | **Arrhythmias** | **Type of SQT** | **Drugs** | **Side effects** | **outcome** | **QTc before drug (ms)** | **QTc on drugs**  **(ms)** | **Follow-up**  **Days** |
| --- | --- | --- | --- | --- | --- | --- | --- | --- | --- | --- | --- | --- | --- | --- |
| **1** | El-Battrawy  2018 | F | - | - | - | Palpitation | None | SQTS | Bisoprolol | None | No effect on QT interval | 320 | 335 | 3073 |
| **2** |  | M | - | - | - | Syncope, Palpitations | Atrial fibrillation | - | Bisoprolol | None | Atrial fibrillation on going  Slight effect on QT interval | 272 | 330 | 5005 |
| **3** | Villafane  2013 | F | KCNQ1 | V141M | I_Ks_ | None | AF | SQTS 2 | Sotalol plus propafenone | None | Recurrence of AF | - | - | 2130 |
| **4** |  | F | KCNJ2 | M301K | I_K1_ | None | AF | SQTS 3 | Flecainide | None | Recurrence of AF | - | - | 2130 |
| **5** |  | M | - | - | - | None | AF | SQTS | Propafenone plus digoxin | None | Recurrence of AF | 355 | 355 | 2130 |
|  |  |  |  |  |  |  |  |  | Dofetilide plus digoxin | None | Prolongation of QT Interval  Return to sinus rhythm | - | 380 |  |
| **8** | Guistetto  2011 | F | KCNH2 | - | I_Kr_ | - | - | SQTS1 | Amiodarone | None | No effect on QT interval | 300 | 284 | 1920 |
|  |  |  |  |  |  |  |  |  | Sotalol | None | No effect on QT interval | 287 | 287 |  |
| **9** |  | M | KCNH2 | - | I_Kr_ | - | - | SQTS1 | Sotalol | None | No effect on QT interval | 283 | 266 | 1920 |
| **10** |  | F | KCNH2 | - | I_Kr_ | - | AF | SQTS1 | Disopyramide | None | Slight effect on QT interval | 329 | 358 | 1920 |
| **11** |  | F | KCNH2 | - | I_Kr_ | - | AF | SQTS1 | Disopyramide | None | No effect on QT interval | 315 | 333 | 1920 |
| **12** |  | M | - | - | - | None | None | SQTS | Disopyramide | None | Slight effect on QT interval | 338 | 365 | 1920 |
| **13** |  | M | - | - | - | SCD | VF | SQTS | Sotalol | None | Ventricular arrhythmia  No effect on QT interval | 349 | 332 | 1920 |
|  |  |  |  |  |  |  |  |  | Amiodarone plus metoprolol | None | No ventricular arrhythmia recorded | 350 | 400 | 182.5 |
| **14** | Guistetto  2015 | M | KCNH2 | T618I | I_Kr_ | None | Ventricular ectopy | SQTS1 | Sotalol | None | Ventricular bigemini, no effect on QT interval | 300 | 310 | 1770 |
| **15** |  | F | KCNH2 | T618I | I_Kr_ | SCD | nsVTs | SQTS1 | Sotalol | None | No effect on QT interval | 340 | 343 | 1770 |

**Table S1: Details of different Short QT syndrome types treated by other drugs**
